# Can Non-farm Employment Improve Dietary Diversity of Left-Behind Family Members in Rural China?

**DOI:** 10.3390/foods13121818

**Published:** 2024-06-10

**Authors:** Yonghu Zhang, Yifeng Zhang, Tingjin Wang

**Affiliations:** College of Economics and Management, Nanjing Forestry University, Nanjing 210037, China; zyh158@njfu.edu.cn (Y.Z.); tjwang@njfu.edu.cn (T.W.)

**Keywords:** dietary diversity, non-farm employment, rural left-behind family members, rural empty nesters

## Abstract

Rural residents in China are still at risk of malnutrition, and increasing dietary diversity is crucial to improving their health. This study empirically analyzed the impact of non-farm employment on the dietary diversity of rural left-behind family members based on the China Land Economy Survey (CLES) 2020–2021 panel data at the farm and village levels. Dietary diversity was measured using the dietary diversity score (DDS) and the Chinese Food Guide Pagoda Score (CFGPS). The empirical results show that non-farm employment significantly enhances the dietary diversity of rural left-behind household members, including animal food diversity and plant food diversity. This result verifies the altruism phenomenon of non-farm employment in family diet. Mechanism analysis shows that non-farm employment enhances the dietary diversity of rural left-behind family members by increasing the level of family income, Internet accessibility, and family education. Heterogeneity analysis shows that non-farm employment does not enhance the dietary diversity of rural empty nesters and even has a negative impact. This reminds us that the nutritional health of rural empty nesters needs attention in the context of rapid urbanization and aging.

## 1. Introduction

In China, there are significant differences in nutrition levels between urban and rural areas [1], and rural residents are at risk of malnutrition and micronutrient deficiency [2,3,4]. Farmers’ consumption of food is insufficient, including both plant food [5] and animal food [6]. Many rural low-income families’ daily diet is only based on grains, lacking the consumption of vegetables, fruits, meat, dairy products, and other foods [7], resulting in micronutrient deficiency and dietary imbalance.

Plant-based foods are rich in vitamins and minerals that help prevent cardiovascular disease, maintain a healthy weight, and extend life [5,8,9]. It has been shown that an inadequate intake of fruits and vegetables contributes to nearly 3 million deaths from heart disease and stroke each year globally [10]. The consumption of pulses can lower blood cholesterol levels and prevent high blood pressure [11]. The increased consumption of plant-based foods such as fruits, vegetables, and pulses is essential for human health. Animal foods, on the other hand, provide more protein of higher quality and more bioavailable vitamin A, vitamin D3, iron, iodine, zinc, calcium, folic acid, and key essential fatty acids [12] and can provide a wide range of micronutrients that are difficult to obtain in sufficient amounts from plant foods alone [13]. It has been found that the benefits of animal foods for humans are not limited to metabolism but also improve immunity [14]. The consumption of animal foods has significant benefits for the growth and development of children and for improving the health of the elderly. For example, the consumption of dairy products in the elderly reduces the risk of frailty and developing sarcopenia [15], and a moderate meat diet maintains physical functioning in the elderly [16]. Meat and milk provide large amounts of energy and nutrients in bioavailable forms, which have positive effects on the growth and development of children [17,18].

The above studies show that Chinese rural residents should increase their consumption of not only plant foods but also animal foods, and since different food types have different nutritional values for the human body, residents should ensure dietary diversity, which is an important way to improve the nutritional health of residents in rural areas. This study will focus on analyzing the issue of improving the dietary diversity of farmers.

Urbanization is regarded by some scholars as an important path to improve the dietary health of rural residents [3]. At the end of 2023, China’s urbanization rate was 66.16%, an increase of 0.94 percentage points from 2022, and more than 10 million rural residents will move into towns and cities every year. The rapid development of China’s urbanization has brought a lot of non-farm employment opportunities to farm households [19]. Rational farmers reconfigure household labor factors and allocate labor to shift to non-farm sector employment for more gains. In the case of households with outgoing non-farm employment, other members of the household who remain in the countryside for a long time are called rural left-behind family members, and it is worthwhile to conduct a study to see whether the quality of the left-behinds’ diets will be improved by the impact of the non-farm employment of family members.

Previous studies have examined the impact of non-farm employment on the food consumption and nutritional intake of rural households from different perspectives. Ma (2022) focused on the carbon footprint perspective to analyze the impact of non-farm employment on the total amount and structure of household food consumption and obtained the conclusion that non-farm employment is positively associated with household food consumption [20]. Bai (2024) analyzed the impact of non-farm employment and agricultural production activities on improving household dietary diversity in Asia and empirically confirmed that non-farm employment is positively associated with household dietary diversity [21]. However, the study did not reveal the welfare impacts on those left behind in rural households. Min et al. (2019) explored the impacts of migration on the food consumption and nutritional intake of rural household members left behind but, however, concluded that the economic rewards of farmers’ migration did not promote the food consumption of those left behind in the household and also led to a reduction in the nutritional intake of the household members left behind [22]. The results of Min’s study deserve further analysis and discussion due to the high degree of linkage between non-farm employment and migration, with non-farm employment personnel usually leaving rural areas and turning to urban areas for employment opportunities. The present study, which also focuses on the factors influencing the welfare of rural left-behind household members, aims to further illustrate the heterogeneity of the effects of non-farm employment on the dietary diversity of left-behind household members.

Based on the existing literature, there are several areas of improvement that can be made at this time. First, in terms of farmers’ diets, there is a scarcity of the literature focusing on the welfare impacts of non-farm employment on members of rural households left behind. Whether non-farm employment can promote the dietary diversity of rural left-behind family members needs to be clarified. Second, what is the mechanism of the impact of non-farm employment on the dietary diversity of rural left-behind family members? There are few studies on the mechanism in the existing literature. Third, along with the accelerated pace of urbanization, rural aging is becoming increasingly serious [23,24]. The phenomenon of empty nesters has become increasingly prominent [25,26,27]; when the rest of the family labor force shifts to non-farm sector employment and only the elderly remain in the family, whether non-farm employment can increase the dietary diversity of the empty nesters and improve their nutritional level needs to be paid attention to.

Accordingly, the main contributions of this study lie in the following points: (1) By empirically analyzing the impact of non-farm employment on the dietary diversity of rural left-behind family members, including animal food diversity and plant food diversity, the instrumental variable approach is used to address the potential endogeneity problem. (2) The issue of the mechanism of the impact of non-farm employment on the dietary diversity of rural left-behind family members is further explored, and it is found that household income increase, Internet accessibility, and improved family education are important mediating mechanisms. (3) Analyzing the heterogeneity of non-farm employment affecting the dietary diversity of rural empty nesters from the perspective of the structure of left-behind members.

The subsequent parts of this paper are organized as follows: Section 2 is the Theoretical Analysis and Research Hypothesis; Section 3 is the Materials and Methods; Section 4 is the Results and Discussion; and Section 5 is the Conclusions and Recommendations.

## 2. Theoretical Analysis and Research Hypotheses

Altruism refers to an individual’s tendency to consider the well-being of others in his or her decision-making and to provide help and support to others to the extent that he or she is able to do so, which is particularly prominent and prevalent in family relationships [28]. In families, individuals voluntarily and unconditionally transfer resources to improve the well-being of family members [29]. Altruistic behavior among family members may be reinforced by increased income from non-farm employment.

Non-farm employment often implies a shift of rural labor to sectors with higher output, which usually leads to an increase in income [30,31]. Increased income can increase consumption capacity [32], including food consumption [33]. Based on the altruism prevalent in the household, farmers’ employment in the non-farm sector strengthens the economic support for the left-behind members within the household by increasing their income [34], which improves the nutritional status of the left-behind members of the household in terms of food availability and dietary diversity.

According to the theory of income elasticity, as income increases, people’s consumption of food does not only increase in quantity but also changes in quality and variety [35]. Animal-based foods (e.g., meat, dairy products, etc.) are often perceived as high-quality foods by rural residents, and these foods are usually more costly than plant-based foods (e.g., vegetables, cereals, etc.). As incomes from non-farm employment increase, rural households may increase their consumption of more expensive animal foods while also increasing their consumption of various types of plant foods in the pursuit of a more balanced dietary structure.

Therefore, non-farm employment increases household income and promotes rural residents to consume a wider variety of foods, including both animal and plant-based foods, to improve dietary diversity and nutrition levels.

Farmers’ demand for information will increase significantly as they transition from the agricultural sector to the non-farm sector [36]. Rural families need to improve the accessibility of the Internet to facilitate work and daily communication [37,38]. The demand for the Internet by left-behind family members due to emotional needs will also increase significantly [39]. As an important channel for information dissemination, the Internet has greatly improved the efficiency and range of people’s access to information [40]. By using the Internet, rural families can more easily obtain information about healthy diet and purchase channels [41]. Family members can learn and share healthy diet information through the Internet, which promotes the formation of good food consumption habits. On the other hand, the use of the Internet can increase the frequency of contact between parents and children and further promote closer intergenerational relationships [42]. Non-farm employees can more efficiently provide intergenerational support to their parents or children through the Internet [43]. China’s e-commerce has broken the restriction of remote rural location, and farmers can buy more diversified and high-quality food products through e-commerce [44].

Non-farm-employed individuals, while working outside, come into contact with more information and different values, including perceptions of education [45]. These experiences may change their views on education, realizing the importance of education in enhancing personal capabilities and changing destinies. Therefore, they tend to pay more attention to the education of the younger generation. Non-farm employment means interaction with a broader social network [46,47], accessing education resources in urban environments. Through these social networks, rural families can obtain information and resources related to education, further promoting intergenerational educational support. Previous studies have also shown that non-farm employment can promote the improvement in family education levels [48]. With the improvement in family education levels, the awareness of healthy eating and diet is also likely to increase among the educated. Based on altruism within the family, left-behind family members in rural areas will also gain information about healthy eating through the transfer of knowledge from the educated, leading them to pay more attention to nutritional value in their food choices and enhancing dietary diversity.

Accordingly, the following hypotheses were formulated in this study:

**Hypothesis** **1.** *Non-farm employment can improve the dietary diversity of rural left-behind family members.*

**Hypothesis** **2.** *Non-farm employment can improve the level of family income, family Internet accessibility, and the level of family education, thereby increasing the dietary diversity of rural left-behind family members.*

## 3. Materials and Methods

### 3.1. Data Source

The data of this study are from the panel data of farmers and villages in the “China land economy survey” (CLES) of Nanjing Agricultural University from 2020 to 2021. The probability proportionate to size sampling (PPS) method is adopted for data sampling. A total of 26 survey districts and counties are selected from 13 prefecture-level cities in Jiangsu Province, 2 sample towns are selected from each district and county, 1 administrative village is selected from each township, and 50 farmers are randomly selected from each village. The survey covers 26 districts and counties in Jiangsu Province, with a total of 2600 households. This study focused on the relationship between non-farm employment and the dietary diversity of rural families, processed the missing and abnormal values of related variables, and finally obtained 4892 samples.

**Figure 1:**
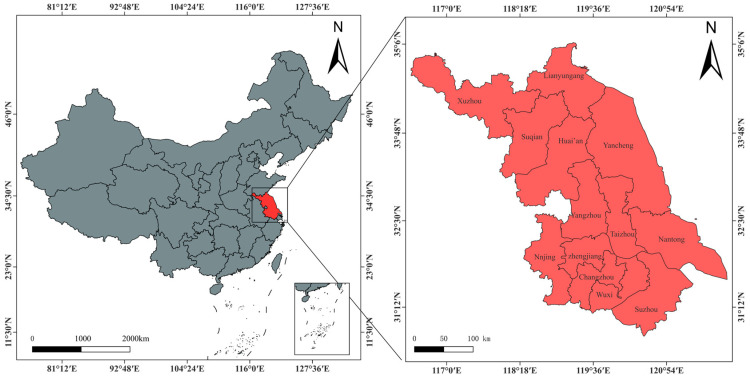
Study area.

In 2023, among the permanent resident population in Jiangsu Province, the population living in urban areas was 63.98 million, an increase of 610,000 over the previous year, and the population living in rural areas was 21.28 million, a decrease of 500,000. By the end of 2023, the urbanization rate of permanent residents in Jiangsu Province exceeded 75%. The increase in urban population means the growth of non-farm employment opportunities. More and more farmers are seeking better employment opportunities in cities and towns. The economic differences within Jiangsu Province can reflect the characteristics of economic differences in western, central, and eastern China [49], so it is representative to select Jiangsu Province as the research area. The study area is shown in Figure 1.

### 3.2. Variable Design and Descriptive Statistics

#### 3.2.1. Dependent Variable

Dietary diversity. Referring to previous studies [21,50,51] and the Food and Agriculture Organization (FAO) classification of food products [52], in order to take into account the characteristics of the Chinese diet and data availability, this study used the types of food consumed by left-behind household members in the past week to measure dietary diversity, using nine food groups, including meat (pork, mutton, beef, poultry), eggs, fish and other aquatic products, milk, cereals (rice, flour, and maize), potatoes, legumes, vegetables, and fruits. Left-behind household members were given a score of 1 if they had consumed a food category in the past week or 0 if they had not consumed that food category, with the total score being the dietary diversity score.

Meanwhile, in order to examine the consumption of food from different sources by left-behind family members, this study further divided the nine food groups into animal food and plant food, and the animal food included four food groups: meat, eggs, fish and other aquatic products, and milk. Plant foods included five food groups: cereals, potatoes, legumes, vegetables, and fruits. The animal food diversity score and plant food diversity score were calculated in the same way as the dietary diversity score.

Chinese Food Guide Pagoda Score (CFGPS). The “Dietary Guidelines for Chinese Residents (2022)” is directed by the National Health and Wellness Commission of the People’s Republic of China and compiled by the Chinese Nutrition Society. The CFGPS is a graphical representation of the principles of a balanced diet translated into the amounts and proportions of each food group according to the guidelines and recommendations of the “Dietary Guidelines for Chinese Residents (2022)”. The CFGPS indicates the recommended range of the daily intake of each food group per adult over a period of time at the energy requirement level of 1600~2400 kcal.

In order to further illustrate the impact of non-farm employment on improving the dietary diversity of rural left-behind family members and ensure the robustness of the conclusions, the CFGPS was introduced by referring to previous studies [53,54]. The CFGPS estimates the difference between the residents’ diet and the recommended diet. This study summarizes the scores of nine food categories, including meat, eggs, fish and other aquatic products, milk, cereals, potatoes, beans, vegetables, and fruits. If the residents’ intake of a certain type of food is within the recommended range, the food score will be assigned as 1. If the intake is between 50% of the lower limit of the recommended range and 150% of the upper limit of the recommended range, the food score will be assigned as 0.5, and the rest will be assigned as 0. The CFGPS is the sum of all food category scores. The higher the score of the CFGPS, the better the dietary diversity and balance of residents. The specific score estimation method and recommended range are shown in Table 1. It should be noted that the unit of measurement of milk by the CLES is bottle. The capacity of most bottled milk in the Chinese market is about 250 mL. This study estimates the milk intake based on this.

#### 3.2.2. Key Independent Variable

Non-farm employment. Referring to previous research [20,55], this variable is measured by the number of family members with non-farm employment. 

#### 3.2.3. Mechanism Variable

Family income level. This study involves two aspects: one is real income, which is measured by annual family income (logarithm); the second is perceived income, which is measured by the satisfaction of family life affluence (satisfaction with housing area, disposable income, etc.) [56]. The combination of real income and perceived income can more comprehensively assess the level of family income. Real income reflects the family’s actual economic situation, while perceived income reflects the family’s subjective feelings about its economic situation.

Family Internet accessibility. Internet accessibility refers to the ability of individuals or families to easily and reliably access the Internet [57]. This study uses two variables: the number of smart phones and the number of computers with Internet access.

Family education level. This study involves two aspects. One is the number of family higher education personnel. If the family members in the sample have more than 12 years of education, they are defined as higher education personnel. The second is education expenditure, which is measured by the total annual expenditure on education (logarithm). The number of family higher education personnel provides an intuitive understanding of the family’s overall education background, and the education expenditure reflects the family’s emphasis on education. By using these two variables, we can more accurately describe the education status of a family.

#### 3.2.4. Instrumental Variable

The proportion of non-farm employment households in the village. In order to address potential endogeneity issues arising from measurement errors, reverse causality, or omitted variables when studying the impact of non-farm employment on the dietary diversity of rural left-behind family members, the instrumental variable ‘the proportion of non-farm employment households in the village’ was introduced to address the issue, measured by the proportion of households in non-farm employment in the sample at the village level. Based on the theory of peer effects [58], individuals are not only influenced by their own characteristics but also by other individuals within the community. The proportion of non-farm employment households in the village reflects the overall tendency and atmosphere of non-farm employment in that village, which is related to non-farm employment at the household level. Therefore, the proportion of non-farm employment households in the village meets the requirement of instrumental variable relevance. Referring to the previous literature [59], the proportion of non-farm employment households in the village is a village-level variable, which is at a different level of observation from household-level non-farm employment and does not directly influence household dietary diversity, meeting the requirement of instrumental variable exogeneity. Therefore, choosing this instrumental variable is reasonable.

#### 3.2.5. Control Variable

Based on research questions and the related literature [20,21,22,60], this study selects three aspects of farmers’ personal characteristics, family characteristics, and village characteristics as control variables. Farmers’ personal characteristics include age, age^2^ (divided by 100), gender, education, cadre, and health. Family characteristics include contracted land area and the number of people eating at home. The distance from the village committee to the nearest highway entrance is selected as the village feature. See Table 2 for specific variables and descriptive statistics.

### 3.3. Model

In order to measure the impact of non-farm employment on the dietary diversity of rural left-behind family members, this paper constructed the following model:

$$Y_{i}=\beta_{0}+\beta_{1}X_{i}+\beta_{2}Z_{i}+\gamma_{p}+\delta_{t}+\varepsilon_{i}$$

where, $Y_{i}$ represents the dietary diversity of the left-behind family members of farmers, i represents different sample farmers, p represents the region, the explained variable $Y_{i}$ represents the dietary diversity of the left-behind family members of the i farmer, the core explanatory variable $X_{i}$ represents the non-farm employment of the i farmer’s family, and $Z_{i}$ represents the set of control variables, including control variables such as respondents’ age, age square (divided by 100), gender, education level, cadre status, health level, family contracted land area, the number of people eating at home, and the distance from the village committee to the nearest highway entrance; $\gamma_{p}$ is the fixed effect of provinces, $\delta_{t}$ is the time fixed effect, and $\varepsilon_{i}$ is the random disturbance term.

## 4. Results and Discussion

### 4.1. Benchmark Regression

Table 3 reports the baseline regression results. Model (1) is the effect of non-farm employment on the dietary diversity of animal foods of rural household members left behind. Model (2) is the effect of non-farm employment on the dietary diversity of plant-based foods of rural household members left behind. Model (3) is the effect of non-farm employment on the dietary diversity of rural left-behind household members. Models (1)–(3) all incorporate control variables and control for time fixed effects and area fixed effects.

The results of Models (1)–(3) show that non-farm employment has a significant positive effect on the dietary diversity of animal food, dietary diversity of plant food, and dietary diversity of rural left-behind household members. Non-farm employment increased the dietary diversity of rural left-behind household members. The results of Bai et al. (2024) illustrated that non-farm employment was significantly and positively associated with household dietary diversity [21], while this study analyzed the effect of non-farm employment on the dietary diversity of rural left-behind household members and considered the nutrient intake of animal food and plant food separately and also arrived at a significant positive association between non-farm employment and the dietary diversity of rural left-behind family members, which enriched research in relevant fields.

### 4.2. Endogenous Processing

Given that the benchmark model may have potential endogeneity problems due to measurement error, omitted variables, reverse causality, etc., this study introduces an instrumental variable (the proportion of non-farm employment households in the village) to correct for the endogeneity problem. Table 4 presents the results of the two-stage regression of the instrumental variable approach.

In phase 1, the coefficients of the instrumental variables were significant at the 1% statistical level, and the F-statistic was greater than the critical empirical value of 10, rejecting the weak instrumental variables hypothesis. In phase 2, non-farm employment at the 1% significance level enhanced the dietary diversity of rural left-behind family members and also significantly enhanced the diversity of animal food and plant food. This verifies Hypothesis 1.

### 4.3. Robustness Test

To verify the robustness of the conclusion, this study introduced the Chinese Food Guide Pagoda Score (CFGPS) as the dependent variable for regression analysis. Table 5 shows the regression results of non-farm employment on the CFGPS of rural left-behind family members. The results show that on the basis of correcting the endogenous problem, non-farm employment significantly increases the CFGPS of rural left-behind family members. This once again verified the role of non-farm employment in promoting the dietary diversity of rural left-behind family members.

### 4.4. Impact Mechanism Testing

Based on the assumptions and empirical findings in the previous section, this part will reveal the intrinsic mechanism of non-farm employment’s effect on the dietary diversity of rural left-behind family members from the three perspectives of family income level, Internet accessibility, and education level. Table 6 shows the test results of the influence mechanism of the rural household income level, Internet accessibility, and the education level.

Models (1) and (2) demonstrate the impact of non-farm employment on rural household income levels. The results show that non-farm employment enhances the annual income of rural households and the satisfaction of family life affluence, and their estimated coefficients are significant at the 1% statistical level. The result illustrates that non-farm employment boosts the income level of rural households from both real and perceived income perspectives. As the household income level increases, the food consumption of left-behind family members will increase, and dietary diversity will be enhanced.

Models (3) and (4) demonstrate the impact of non-farm employment on rural household Internet accessibility. The results show that non-farm employment enhances rural household Internet accessibility, as evidenced by an increase in the number of household smartphones and Internet-accessible computers, whose estimated coefficients are both significant at the 1% statistical level. With the increase in household Internet accessibility, firstly, non-farm employment can give intergenerational support to their left-behind relatives through the Internet, thus promoting their food consumption and increasing dietary diversity; secondly, through the use of the Internet, farmers’ dietary nutritional knowledge increases, and they pursue a balanced and diversified diet more.

Models (5) and (6) demonstrate the impact of non-farm employment on the level of education of rural households. The results show that non-farm employment boosts the number of tertiary education personnel and household education expenditures in rural households, and their estimated coefficients are both significant at the 1% statistical level. The result illustrates that non-farm employment promotes the level of education of rural households in terms of both the overall educational background of the household and the importance attached to education. As the level of household education increases, tertiary education personnel indirectly influence the food consumption concepts of all household members through their own knowledge accumulation, promoting dietary diversity.

The results of the above tests confirm that non-farm employment will have an impact on the dietary diversity of rural left-behind household members by increasing the level of household income, Internet accessibility, and education. Hypothesis 2 was verified.

### 4.5. Heterogeneity Analysis

Based on the growing phenomenon of rural empty nesters, this study analyzed the heterogeneity of non-farm employment affecting the dietary diversity of rural empty nesters. Table 7 demonstrates the results of the heterogeneity analysis. Generally, empty nesters are those who do not have children to take care of them and live alone or in couples, so this study analyzes them from two aspects: empty nesters living together and empty nesters living alone.

In this study, cohabiting empty nesters refer to rural households with only two permanent residents over the age of 60, who can be either husband and wife or companions living in partnership. Empty nesters living alone are defined as those who have only one permanent resident in a rural household who is 60 years of age or older.

Models (1) to (6) all show that the effect of non-farm employment on dietary diversity is not significant for either cohabiting empty nesters or single nesters, and the coefficients of non-farm employment are even all negative in the case of single nesters, which is a surprising result. The study of Min et al. (2019) also obtains the conclusion that migration leads to a reduction in the nutrient intake of family members who are left behind [22]. According to the results of this study, the important factor of negative correlation is the structure of left-behind family members, and when the structure of left-behind family members is empty nesters, non-farm employment does not have a positive effect on the dietary health of the empty nester population.

Against the backdrop of rapid urbanization, many rural elders have been left alone in the countryside, and the income-generating effects of the non-farm employment of family members, the convenience of the Internet, and the increased educational level of family members have not theoretically enhanced the dietary diversity of empty nesters. This is a wake-up call that in rural China, when there are only elderly people in the home, especially when there is only one elderly person, more time for care and companionship may be more important for the dietary health of the elderly.

## 5. Conclusions and Recommendations

This study is based on the “China Land Economic Survey” (CLES) 2020–2021 panel data of rural households and village-level data. It empirically analyzes the impact of non-farm employment on the dietary diversity of rural left-behind family members and draws the following important conclusions. Firstly, non-farm employment improves the dietary diversity of rural left-behind family members, including both animal-based and plant-based food diversity. To ensure the robustness of the conclusions, this study replaces the dependent variable with the Chinese Food Guide Pagoda Score (CFGPS) for regression analysis. The results indicate that non-farm employment significantly increases the CFGPS of rural left-behind family members, once again demonstrating the significant positive impact of non-farm employment on improving the dietary diversity of rural left-behind family members and confirming the altruistic phenomenon of non-farm employment in family food consumption. Accordingly, Hypothesis 1 is validated. Secondly, mechanism analysis shows that non-farm employment enhances the dietary diversity of rural left-behind family members by improving the income level, Internet accessibility, and education level of rural households. Accordingly, Hypothesis 2 is validated. Heterogeneity analysis shows that non-farm employment does not significantly enhance the dietary diversity of rural empty-nest elderly individuals and may even have a negative impact on them. This to some extent indicates that the dietary habits of rural empty-nest elderly individuals are not only related to household income and nutritional awareness but also require attention to their spiritual life to address the issue of insufficient time for caring for the elderly caused by their children’s non-farm employment. Based on this, this study proposes the following recommendations. 

Firstly, promote urbanization construction, provide more non-farm employment opportunities to farmers, and transfer surplus labor from rural areas. Relevant departments are suggested to strengthen non-farm vocational education or training for farmers to ensure smooth employment after training.

Secondly, enhance the construction of information infrastructure in rural areas, including Internet coverage, and the establishment of e-commerce service stations. Promote smartphones and improve the information acquisition capabilities of rural residents. Provide training and support to help farmers master the skills of using the Internet and e-commerce platforms and enhance their digital literacy.

Thirdly, increase investment in rural education and improve the quality of education and the allocation of educational resources. Expand the scope of education subsidies to reduce the educational burden on rural families.

Fourthly, care for rural empty-nest elderly individuals. Establish elderly canteens to provide professional nutritional meal services. Set up more entertainment activities and related venues to enrich the lives of empty-nest elderly individuals. Strengthen community volunteer services, organize volunteers to visit empty-nest elderly individuals regularly, care for their lives and mental health, and address the issue of insufficient time for caring for the elderly caused by their children’s non-farm employment. Promote the knowledge of Internet use among rural empty-nest elderly individuals and enhance intergenerational emotional communication by increasing the Internet usage rate among them.

Finally, this study also has some limitations. This study considers the heterogeneity of the structure of rural left-behind family members but is limited by data and only analyzes the heterogeneity of the phenomenon of empty-nest elderly individuals. In addition, the survey sample is from Jiangsu Province, China. Although Jiangsu Province as a research sample has a certain representativeness, future research can be expanded to more extensive areas to draw conclusions on the heterogeneity between different regions.

## Figures and Tables

**Table 1:** Method of estimating Chinese Food Guide Pagoda Score.

Food Group	Consumption (g)	Recommended Scope (g)
Grains		200–300
Score = 1	200–300	
Score = 0.5	100–200 or 300–450	
Score = 0	Else	
Potatoes		50–100
Score = 1	50–100	
Score = 0.5	25–50 or 100–150	
Score = 0	Else	
Beans		25–35
Score = 1	25–35	
Score = 0.5	12.5–25 or 35–52.5	
Score = 0	Else	
Vegetables		300–500
Score = 1	300–500	
Score = 0.5	150–300 or 500–750	
Score = 0	Else	
Fruits		200–350
Score = 1	200–350	
Score = 0.5	100–200 or 350–525	
Score = 0	Else	
Meat		40–75
Score = 1	40–75	
Score = 0.5	20–40 or 75–112.5	
Score = 0	Else	
Eggs		40–50
Score = 1	40–50	
Score = 0.5	20–40 or 50–75	
Score = 0	Else	
Aquatic products		40–75
Score = 1	40–75	
Score = 0.5	20–40 or 75–112.5	
Score = 0	Else	
Milk		300–500
Score = 1	300–500	
Score = 0.5	150–300 or 500–750	
Score = 0	Else	

**Table 2:** Descriptive statistics of variables.

Variable Type	Variable Definition	Variable Description and Assignment	Mean Value	Standard Deviation
Dependent variable	Dietary diversity	Dietary diversity score	7.261	1.542
	Animal-based food diversity	Animal-based food diversity score	3.061	0.946
	Plant-based food diversity	Plant-based food diversity score	4.200	0.920
	Chinese Food Guide Pagoda	Chinese Food Guide Pagoda score	2.990	1.154
Independent variable	Non-farm employment	Number of family non-farm employment	1.631	1.249
Mechanism variable	Income quality	Annual household income (logarithmic)	10.992	1.157
		Satisfaction with family life prosperity (housing area, disposable income, etc.)	3.622	0.917
	Internet accessibility	Number of home smartphones	2.484	1.675
		Number of computers with Internet access at home	0.603	0.901
	Education level	Number of higher education talents	0.493	0.752
		Annual education expenditure (logarithmic)	3.338	4.414
Control variable	Age	Age (years)	61.575	11.388
	Age^2^	Age squared term/100	39.211	13.233
	Gender	Male = 1; Female = 0	0.715	0.452
	Education	Education level (years in school)	7.032	3.965
	Cadre	Do you have a position in this village? Yes = 1, No = 0	0.152	0.359
	Health	Self-perceived health status (1 = loss of labor ability; 2 = poor; 3 = moderate; 4 = good; 5 = excellent)	3.974	1.070
	Land scale	Contracted land area (hectare)	0.189	0.822
	The number of people eating at home	The number of people eating at home in the past week	2.988	1.816
	Distance	The distance from the village committee to the nearest highway entrance (kilometers)	13.030	16.209
Instrumental variable	The proportion of non-farm employment households in the village	The proportion of households in non-farm employment in the sample at the village level	0.777	0.102

**Table 3:** Benchmark regression results.

	(1)	(2)	(3)
	Animal-Based Foods	Plant-Based Foods	Dietary Diversity
Non-farm employment	0.033 ***	0.027 **	0.060 ***
	(0.012)	(0.012)	(0.021)
Age	−0.013	0.018 **	0.005
	(0.008)	(0.008)	(0.014)
Age^2^	0.008	−0.018 **	−0.009
	(0.007)	(0.007)	(0.012)
Gender	−0.004	0.002	−0.001
	(0.031)	(0.031)	(0.050)
Education	0.038 ***	0.027 ***	0.064 ***
	(0.004)	(0.004)	(0.006)
Cadre	0.123 ***	0.062 *	0.185 ***
	(0.033)	(0.035)	(0.056)
Health	0.067 ***	0.044 ***	0.111 ***
	(0.014)	(0.013)	(0.022)
Land scale	0.020	0.014 *	0.034 *
	(0.013)	(0.008)	(0.020)
Number of people eating at home	0.086 ***	0.061 ***	0.147 ***
	(0.024)	(0.018)	(0.042)
Distance	−0.001	−0.001	−0.001
	(0.001)	(0.001)	(0.002)
Time fixed effects	YES	YES	YES
Regional fixed effects	YES	YES	YES
_cons	2.666 ***	3.210 ***	5.876 ***
	(0.262)	(0.267)	(0.459)
N	4892	4892	4892
R^2^	0.148	0.082	0.130

Standard errors in parentheses; * *p* < 0.1, ** *p* < 0.05, *** *p* < 0.01; the following tables are the same.

**Table 4:** Analysis results of instrumental variables.

Variable	Animal-Based Foods		Plant-Based Foods		Dietary Diversity	
	Phase 2	Phase 1	Phase 1	Phase 2	Phase 1	Phase 2
Non-farm employment	0.253 *** (0.089)		0.377 *** (0.092)		0.630 *** (0.157)	
Instrumental variable		2.240 *** (0.234)		2.240 *** (0.234)		2.240 *** (0.234)
Control variable	YES	YES	YES	YES	YES	YES
Time fixed effects	YES	YES	YES	YES	YES	YES
Regional fixed effects	YES	YES	YES	YES	YES	YES
F-statistic	88.214		88.214		88.214	
N	4892	4892	4892	4892	4892	4892

Standard errors in parentheses; *** *p* < 0.01.

**Table 5:** Robustness test results.

	OLS	2SLS	
Variable	(1) CFGPS	(2) Phase 2	(3) Phase 1
Non-farm employment	0.017	0.295 ***	
	(0.016)	(0.114)	
Instrumental variable			2.240 ***
			(0.234)
Control variable	YES	YES	YES
Time fixed effects	YES	YES	YES
Regional fixed effects	YES	YES	YES
R^2^	0.062		
F-statistic		88.214	
N	4892	4892	4892

Standard errors in parentheses; *** *p* < 0.01.

**Table 6:** The results of the mechanism analysis.

	Income Level		Internet Accessibility		Education Level	
Variable	(1) Income	(2) Satisfaction	(3) Smartphones	(4) Computers	(5) Higher Education	(6) Education Expenditure
Non-farm employment	0.274 ***	0.053 ***	0.483 ***	0.129 ***	0.203 ***	0.246 ***
	(0.021)	(0.011)	(0.026)	(0.011)	(0.010)	(0.078)
Control variable	YES	YES	YES	YES	YES	YES
Time fixed effects	YES	YES	YES	YES	YES	YES
Regional fixed effects	YES	YES	YES	YES	YES	YES
N	2101	4874	4828	4892	4892	4771
R²	0.377	0.094	0.359	0.188	0.220	0.258

Standard errors in parentheses; *** *p* < 0.01.

**Table 7:** Results of heterogeneity analysis.

	Animal-Based Foods		Plant-Based Foods		Dietary Diversity	
Variable	(1) Couple	(2) Alone	(3) Couple	(4) Alone	(5) Couple	(6) Alone
Non-farm employment	0.025	−0.010	−0.000	−0.031	0.025	−0.041
	(0.019)	(0.052)	(0.021)	(0.055)	(0.034)	(0.088)
Control variable	YES	YES	YES	YES	YES	YES
Time fixed effects	YES	YES	YES	YES	YES	YES
Regional fixed effects	YES	YES	YES	YES	YES	YES
N	1244	313	1244	313	1244	313
R^2^	0.104	0.197	0.071	0.126	0.096	0.173

## Data Availability

The data underlying the results presented in this study are all available. The data presented in this study are available on request from the corresponding author. The data are not publicly available due to privacy.
